# A model of mobile robots in networks with resolvability properties

**DOI:** 10.1371/journal.pone.0325565

**Published:** 2025-06-17

**Authors:** Carlos Camacho Campos, José Carlos Camacho Moreno, Dorota Kuziak, Zahid Raza, Ismael G. Yero

**Affiliations:** 1 Departamento de Ingeniería Civil, Universidad de Cádiz, Algeciras Campus, Spain; 2 Departamento de Matemáticas, Universidad de Cádiz, Algeciras Campus, Spain; 3 Departamento de Estadística e Investigación Operativa, Universidad de Cádiz, Algeciras Campus, Spain; 4 Department of Mathematics, University of Sharjah, Sharjah, United Arabs Emirates; European Commission, ITALY

## Abstract

A model for mobility of robots keeping the property of uniquely recognizing the vertices of a given network is considered in this work. This is made in order to detect failures or intruders, by means of dynamic vectors of distances to the set of mobile robots. We consider the smallest set of robots that can be placed in a set of nodes of a network that forms a resolving set, which is a structure of a graph such that it uniquely recognizes all the vertices of the graph by using distances. We are then focused on allowing such robots to move from one vertex to another adjacent one, through the edges of the whole graph. At each performed movement we require that the new set of covered nodes forms a resolving set. This process allows the robots to recognize all the vertices of the graph, independently on the position in which they are located. In this sense, the notion of mobile metric dimension is introduced in this article, and the study of its primary combinatorial properties is initiated. We relate this parameter with the classical metric dimension and the resolving number of graphs and compute its value for several graph classes.

## 1 Introduction

The location of intruders or failures in networks, as well as, the mobility of robots keeping a certain property on the movements of the robots are typical problems in computer engineering, as well as, in several other scientific areas. In this work, our aim is to consider a mobility situation of robots in a network, while maintaining the property of uniquely recognizing the elements of the network. This situation is studied in order to detect failures or intruders, by means of dynamic vectors of distances to the set of mobile robots.

A resolving set *S* in a graph *G* (subset of vertices of such *G*) represents a structure with the property that each vertex of *G* is uniquely represented by a vector of distances to the vertices of *S*. This uniqueness capability of *S* has been crucial while designing monitoring systems or robot navigation models in networks, which might be somehow understood to be similar to a set of satellites required for GPS to efficiently work. Some considerations on these directions have appeared in [4,18,24]. A natural setting requires then for an optimal (as smallest as possible) number of recognition devices (elements in the resolving set - also called “landmarks”). In addition, the same recognition feature existing in such landmarks in a network can be meaningful while tracing the development of a disease that spreads between communities of people. On the other hand, according to [30], such resolving sets can be used to identify source points of misinformation in networks.

Some other applications of related resolving parameters are known in the literature. Among them, the reader can find applications to pharmaceutical sciences in [21,22]; to pattern recognition and image processing in [27]; to the design of error correcting codes in [1]; and to measuring the privacy in social networks against active attacks in [31]. With respect to this last remarkable application, the recent work [9], published in the reputed journal Scientific Reports, has given a privacy evaluation of several random networks through the use of a linear optimization model. These facts above show the significance of these metric problems in graphs.

Despite the unquestionable usefulness of resolving sets (in several of its variants), there could be some practical settings that would require some improvements. To see this, consider a monitoring system formed by a set of landmarks *S* in a network *G* (clearly, able to identify the vertices of *G* by means of a vector of distances to the vertices in *S*), which would need not only to monitor the vertices of *G*, but also to repair a failure if it appears. This is clearly possible if landmarks are for instance agents that repair the failure points and are carrying out the monitoring devices. In this sense, if a landmark moves its position from one point to the failing one, it could clearly lose the capability of identifying the remaining vertices of the network, and of course, the possibility of detecting other possible failures. A possible solution to this fact could be that of using a fault-tolerant resolving set as the set of landmarks, a notion first described in [17], or that more general one of *k*-resolving sets (first defined in [8]), which can be understood as a resolving set able to tolerate at most k≥1 faults. However, as we shall observe from our deductions, this is indeed not the best solution for the problem we approach here.

A better possible solution to the problem mentioned above, is that all the time the set of landmarks will have the possibility of uniquely recognizing the vertices of the network, despite the fact that a landmark of the resolving set would have been required to move its location from one point to another one (probably because of a failure in some point or any other specific situation out of control of the monitoring system itself). This gives a step forward introducing our proposal for a mobile navigation model.

Formally, we shall consider a set of robots standing in a set of nodes of a network that forms a resolving set, which is (as already mentioned) a structure of a graph that uniquely recognizes all the vertices in the graph by using (a vector of) distances. We are then focused on allowing such robots to move from one occupied vertex to another adjacent not occupied one, through the edges of the whole graph, and requiring that, at each performed movement, the new set of covered nodes forms a resolving set. This process allows the robots to recognize all the vertices of the graph, independently on the position in which they are located. With the purpose of optimizing the resources, it is desirable to have the property of using the smallest possible number of robots to be placed in the given network.

We consider G=(V,E) is a connected, undirected and simple graph without loops and multiple edges. The set of vertices and of edges of *G* shall be written as *V*(*G*) and *E*(*G*), respectively. Given two vertices u,v∈V(G), the *distance* between *u* and *v* is the length of a shortest *u*,*v*-path, and is denoted by $d_{G}(u,v)$. The two vertices *u*,*v* are *resolved, identified* or *recognized* by a vertex x∈V(G) if $d_{G}(u,x)\neq d_{G}(v,x)$. It is also said that *x resolves, identifies* or *recognizes* the pair of vertices *u*,*v*. Moreover, by R{x,y} we mean the set of all vertices of *G* that resolves the pair *x*,*y* (note that *x*,*y* are in R{x,y}). In this sense, a set of vertices S⊂V(G) is a *resolving set* of *G* if any two vertices in *V*(*G*) are resolved by a vertex of *S*. A resolving set with the smallest possible cardinality is called a *metric basis*. The cardinality of a metric basis in *G* is the *metric dimension* of *G*, denoted by dim(G).

The concepts above were independently and first presented in [16,29] for the graph theory area. However, it remained weakly and naively investigated until the beginning of this century, when the article [4] motivated an increasing attention to the parameter. Nowadays, the topic of metric dimension in graphs is a classical one, and a reader can find a rich literature on both theoretical and applied results, as well as, a number of open problems that remain unsolved. Some recent and significant contributions on this topic are [6,7,10,12,14,15,26,28,33]. For more information on metric dimension of graphs and related topics we suggest the recent surveys [25,30].

**Fig 1:**
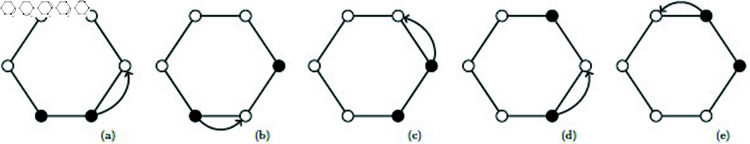
The first five valid resolving moves of a mobile resolving set in the cycle ${\text{C}}_{6}$ from (a) to (e): bolded vertices represent the robots of the mobile resolving set at each step. Notice that in this way, making three cyclic movements more, all the vertices of *C*_6_ shall be visited by valid resolving movements. On the other hand, observe that cyclic movements in the opposite way can be also done with a same result.

Given a resolving set *S* of a graph *G* occupied by a set of robots, and a vertex *v* of *S*, the robot located at the vertex *v* makes a *valid resolving movement*, if it moves from *v* to a neighbor of it (not occupied by another robot), say $v^{\prime }$, such that the new set $S⧵\{v\}\cup \{v^{\prime }\}$ occupied by the robots is also a resolving set of *G*. A valid resolving movement from *v* to $v^{\prime }$ shall be represented as $v⇝v^{\prime }$. Now, we say that a set of vertices S⊂V(G) is a *mobile resolving set* if every vertex of *G* can be visited by at least one of the robots initially placed at the resolving set *S*, in a sequence of valid resolving movements. The *mobile metric dimension* of *G* is the cardinality of a smallest mobile resolving set of *G*, which is denoted by mmd(G). A mobile resolving set of cardinality mmd(G) is called a *mobile metric basis*. From now on, in order to facilitate our exposition, by a robot *x*, we mean a robot that is placed at a vertex x∈V(G) of the graph *G*. We remark the fact that there could be several different sequences of valid resolving movements that will allow the robots placed at a mobile resolving set to visit all the vertices of a graph. Finding such sequences might be a challenging problem, and it is not in the scope of this investigation. In Fig 1 we present a fairly representative example of the concepts above.

Other necessary terminologies and notations we need are as follows. The *Cartesian product* G◻H of the graphs *G* and *H* has vertex set equal to V(G)×V(H). Two vertices $\left(g,h),(g^{\prime },h^{\prime }\right)$ are adjacent in G◻H if either $g=g^{\prime }$ and $h,h^{\prime }$ are adjacent in *H*; or $h=h^{\prime }$ and $g,g^{\prime }$ are adjacent in *G*.

## 2 First basic results

It is clear that any mobile resolving set is also a resolving set. On the other hand, since any set of cardinality |V(G)| is a resolving set of a graph *G*, it can readily observed that a mobile resolving set of such cardinality always exists, which means that the concept is well defined. As a consequence, the following bounds are clear for any connected graph *G* of order *n*, since also the metric dimension of any graph is at least equal to 1.

n−1≥mmd(G)≥dim(G)≥1. (1)

As we next show, an example where the equality in the bound above is achieved is for instance the case of cycle graphs *C*_*n*_ with n≠4, for which it is known that $\dim(C_{n})=2$.

**Proposition 2.1.** *For any cycle C*_*n*_ *with n≥3,*


$$mmd(C_{n})=\{\begin{array}{ll} 3, & if\;n=4,\\ 2, & otherwise. \end{array}$$

*Proof:* For the cycle *C*_3_, it is trivial to check that $\text{mmd}(C_{3})=2$. Now, in the cycle *C*_4_ any metric basis is formed by two adjacent vertices. However, no robot placed at a vertex of such metric bases can make a valid resolving movement. Thus, any mobile resolving set of *C*_4_ cannot be a metric basis of *C*_4_. This leads to $\text{mmd}(C_{4})\geq 3$, which is indeed an equality, since any three vertices of *C*_4_ form a mobile resolving set.

On the other hand, let $C_{n}=v_{0}v_{1}\cdots v_{n-1}v_{0}$ with n≥5. It is well known that $\dim(C_{n})=2$ and that any two adjacent vertices of *C*_*n*_, as well as any two vertices at distance two (when n≥5), form a metric basis. In this sense, we consider the metric basis $S=\{v_{i},v_{i+1}\}$ with i∈{0,…,n−1} and assume the operations with the subindex are done modulo *n*. It can be readily seen that $v_{i+1}⇝v_{i+2}$ represents a valid resolving movement since it produces a new metric basis of *C*_*n*_ with two vertices at distance two. Then, the subsequent $v_{i}⇝v_{i+1}$ is again a valid resolving movement, since it produces again a new metric basis with two adjacent vertices. Thus, by repeating this process, we can find a sequence of valid resolving movements that allows to visit all the vertices of *C*_*n*_. Therefore, $\text{mmd}(C_{n})\leq 2=\dim(C_{n})$, and by (1), the conclusion is deduced. ◻

Following with the notion of vertex transitive graphs (like cycles), we next consider the case of circulant graphs *C*_*n*_(2). A *circulant graph C*_*n*_(*t*) is a graph with vertex set $V(C_{n}(t))=\{1,2,\ldots ,n\}$ and such that two vertices $i,j\in V(C_{n}(t))$ are adjacent if either |i−j|≤t or n−|i−j|≤t. We are focused in this work in the case *t* = 2. The distance formula for any two vertices $i,j\in V(C_{n}(2))$ is

$$d_{C_{n}(2)}(i,j)=\left\{ \begin{array}{cc} \left\lceil \frac{\left|i-j\right|}{2}\right\rceil ; & \text{if}\;|i-j|\leq n/2,\\ \left\lceil \frac{n-|i-j|}{2}\right\rceil ; & \text{if}\;|i-j|>n/2. \end{array}\right.$$ (2)

The metric dimension of circulant graphs has been somehow intensively studied in several articles, from which we remark [2,13,19,20,32]. For the particular case of *C*_*n*_(2), the following was proved in [2,20]. For any integer n≥5,

$$\dim(C_{n}(2))=\left\{ \begin{array}{cc} 4; & \text{if}\;n\equiv 1\;\text{(mod 4)},\\ 3; & \text{otherwise.} \end{array}\right.$$ (3)

By using some computer computations, we have first obtained the following for small values of *n*, that is, $\text{mmd}(C_{5}(2))=\text{mmd}(C_{6}(2))=4$ and $\text{mmd}(C_{7}(2))=3$. We next give the remaining values for larger values of *n*.

**Theorem 2.2.** *For any integer n≥8, $\text{mmd}(C_{n}(2))=\dim(C_{n}(2))$.*

*Proof:* Based on the distance formula (2), we first note that each two vertices of *C*_*n*_(2), other than the pairs 2i+1,n−2(i−1) with 1≤i≤⌊n/4⌋, are resolved by at least one of the vertices $1,2\in V(C_{n}(2))$, unless *n* = 4*k* + 1 for some k≥1, where also each pair of the set of three vertices 2k+1,2k+2,2k+3 are neither resolved by 1 nor by 2 (such facts were already noted in [2,20]). Thus, if we want to show that a set of vertices properly containing 1,2 is a resolving set, then we only need to prove that such pairs are resolved by some extra vertices different from 1 and 2.

Assume first that n≡1 (mod 4), say n=4k+1 for some k≥2. From (3), we know that $\dim(C_{4k+1}(2))=4$. From the proof of such result presented in [2,20], it is known that the set of vertices $S^{\prime }=\{1,2,3,4\}$ is a metric basis for *C*_4*k* + 1_(2). We first claim that also the set S={1,2,4,5} is a resolving set of *C*_4*k* + 1_(2). To see this, observe that due to the symmetry of *C*_4*k* + 1_(2) by using the arguments from the previous paragraph (for the vertices 1,2), the pairs of vertices not resolved by the vertices 4,5 are

(i) the pairs 2i+4,4k+1−2(i−1)+3 with 1≤i≤k−1, and
(ii) each pair of the set of three vertices 2k+4,2k+5,2k+6.

We recall that the set of vertices not resolved by 1,2 are

(iii) the pairs 2j+1,4k+1−2(j−1) with 1≤j≤k−1, and
(iv) each pair of the set of three vertices 2k+1,2k+2,2k+3.

(We relatively abuse the notation and say that the vertex, if it appears in the above sums, *n* + *t* is indeed the vertex *t* for any 1≤t≤n−1).

Now, we observe that from the pairs of vertices in (i) at least one is even. In contrast, the pairs of vertices from (iii) are both odd. This means that such pairs from (i) and (iii) will never coincide. Also, the vertices of (ii) and (iv) are consecutive and different, while the vertices *i* from (i) and *j* from (iii) satisfy that |i−j|≥3. Thus, there is no pair of vertices from (i)-(iv) that appears in two different items. This means that any two vertices of *C*_4*k* + 1_(2) is identified by at least one vertex of S={1,2,4,5}, and so, it is a metric basis since $\dim(C_{4k+1}(2))=4$.

Having these facts in mind, we consider a metric basis *S*_1_ = {1,2,3,4} of *C*_4*k* + 1_(2), and place one robot in each of these vertices. Next, by using the arguments above, we have that the movement 3⇝5 is a valid resolving movement since *S*_2_ = {1,2,4,5} is a metric basis of *C*_4*k* + 1_(2) as shown. Now, the movement 1⇝3 leads to the set *S*_3_ = {2,3,4,5} which, by symmetry, is also a metric basis for *C*_4*k* + 1_(2). Hence, by using this idea, one can make a sequence of analogous valid resolving movements that allows to visit all the vertices of *C*_4*k* + 1_(2). Therefore, *S*_1_ is a mobile resolving set, and so, $\text{mmd}(C_{n}(2))\leq 4=\dim(C_{n}(2))$. The equality is completed by (1).

From now on, we assume n≢1 (mod 4). In such situation, each two vertices of *C*_*n*_(2), other than the pairs 2i+1,n−2(i−1) with 1≤i≤⌊n/4⌋, are resolved by at least one of the vertices $1,2\in V(C_{4k}(2))$. Again, if one wants to show that a set of vertices properly containing 1,2 is a resolving set, then it is necessary to prove that such pairs are resolved by some extra vertices different from 1 and 2.

It is known from [20] that $\dim(C_{n}(2))=3$, and along the proof of such result, it is shown that the set $S^{\prime }=\{1,2,3\}$ is a metric basis for *C*_*n*_(2). We claim that also the set S={1,2,5} is a metric basis for *C*_*n*_(2). Since 1,2 are part of *S*, by the arguments above, we only need to show that pairs of vertices 2i+1,n−2(i−1) are resolved by the vertex 5. We can w.l.g. assume that 2i+1<n−2(i−1). By using the distance formula (2), if *i* = 1, then $d_{C_{n}(2)}(3,5)=1$ and $d_{C_{n}(2)}(n,5)=3$, unless *n* = 8 when $d_{C_{n}(2)}(n,5)=2$. Also, if *i* = 2, then $d_{C_{n}(2)}(5,5)=0$ and $d_{C_{n}(2)}(n-2,5)\neq 0$. In any other case, that is 3≤i≤⌊n/4⌋, it holds,


$$d_{C_{n}(2)}(2i+1,5)=\left\lceil \frac{\left|2i-4\right|}{2}\right\rceil =i-2,$$

On the other hand, if n−2(i−1)−5>n/2, then


$$d_{C_{n}(2)}(n-2(i-1),5)=\left\lceil \frac{n-(|n-2(i-1)-5|)}{2}\right\rceil =\left\lceil \frac{n-(n-2i-3)}{2}\right\rceil =\left\lceil \frac{2i+3}{2}\right\rceil =i+2.$$

Also, if n−2(i−1)−5≤n/2, then by using the fact that i≤⌊n/4⌋ (or equivalently n≥4i), we deduce that


$$d_{C_{n}(2)}(n-2(i-1),5)=\left\lceil \frac{\left|n-2(i-1)-5\right|}{2}\right\rceil =\left\lceil \frac{\left|n-2i-3\right|}{2}\right\rceil \geq \left\lceil \frac{\left|2i-3\right|}{2}\right\rceil >i-2.$$

Thus, we deduce that the vertex 5 resolves every pair 2i+1,n−2(i−1) with 1≤i≤⌊n/4⌋, and so, *S* is a metric basis as claimed.

Similarly to the previous situation, we begin with a set *S*_1_ = {1,2,3} and place a robot in each of these vertices. By the reasons above, we readily see that 3⇝5 is a valid resolving movement since *S*_2_ = {1,2,5} is a metric basis. Now, observe that the movement 2⇝4 leads to the set *S*_3_ = {1,4,5}, which by its symmetry with the set *S*_2_, is also a metric basis of *C*_*n*_(2). Next, by its symmetry with respect to *S*_1_, we have that the set *S*_4_ = {3,4,5}, that can be obtained from *S*_3_ by the movement 1⇝3, is also a metric basis. In this sense, we repeat the process as many times are required, and we hence develop a sequence of valid resolving movements that allows to visit all the vertices of *C*_*n*_(2). Thus, *S*_1_ is a mobile resolving set, and so, $\text{mmd}(C_{n}(2))\leq 3=\dim(C_{n}(2))$. The desired equality again follows by (1), which completes the proof. ◻

The central inequality (mmd(G)≥dim(G)) of (1) can be also strict. The concept of *void vertex* of a graph *G* was introduced in [15] as a vertex which does not belong to any metric basis of *G*. For instance, in a path of at least three vertices, any vertex of degree two is a void vertex. The following result is then obtained.

**Remark 2.3.** *If a graph G has a void vertex, then mmd(G)≥dim(G)+1.*

*Proof:* Since a void vertex *v* does not belong to any metric basis of *G*, in order to visit *v* by a valid movement made by a robot of a mobile resolving set of *G*, it is necessary that such mobile resolving set would have cardinality at least dim(G)+1. Thus, mmd(G)≥dim(G)+1. ◻

The equality in the lower bound above occurs in several situations. That is stated in our next results.

**Remark 2.4.** *If G is a graph with mmd(G)≥dim(G)+1 and that has a metric basis S whose complement induces a connected graph, then mmd(G)=dim(G)+1.*

*Proof:* If the complement of *S* induces a connected graph, then for any vertex u∉S it is clear that $S^{\prime }=S\cup \{u\}$ is also a resolving set. Since a robot placed at this vertex *u* can visit all the vertices of *G* not in *S* by a sequence of resolving movements (because the complement of *S* induces a connected graph), we deduce that $S^{\prime }$ is a mobile resolving set. Thus, $\text{mmd}(G)\leq |S^{\prime }|=\dim(G)+1$, and the equality follows since mmd(G)≥dim(G)+1 by assumption. ◻

The result above covers a large class of graphs, namely, all the graphs having at least one void vertex and a metric basis *S* whose complement induces a connected graph. Some examples of such graphs include for instance trees of order at least three and grid graphs. We next list a few of them.

**Corollary 2.5.** *The following statements holds.*

(i) *For any path P*_*n*_, $\text{mmd}(P_{n})=\left\{ \begin{array}{cc} 1, & \text{if}\;n=2,\\ 2, & \text{if}\;n\geq 3. \end{array}\right.$
(ii) *For any tree T of order at least three, mmd(T)=dim(T)+1.*
(iii) *For any grid graph $P_{r}\;◻\;P_{t}$, $\text{mmd}(P_{r}\;◻\;P_{t})=3$.*

We now consider a useful observation, where we need the following terminology. The *open neighborhood N*_*G*_(*x*) of a vertex *x* is the set of neighbors of *x*, and the *closed neighborhood* of *x* is $N_{G}[x]=N_{G}(x)\cup \{x\}$. Two vertices *x*,*y* of a graph *G* are called *true twins* or *false twins* if they have the same closed or open neighborhood, respectively. A vertex *x* is called a *twin*, if there is another vertex *y* such that *x* and *y* are either true or false twins.

Item (ii) from Corollary 2.5, among other conclusions, allows to observe that using fault-tolerant resolving sets to solve the problem we deal with in this exposition is not the best solution. That is, from Corollary 2.5 (ii), we have mmd(T)=dim(T)+1, while from [17], the existence of fault-tolerant resolving sets in a tree *T* containing a number of vertices significantly larger than dim(T) is already known.

**Remark 2.6.** *If a graph G has a universal vertex which is not a twin vertex, then mmd(G)=dim(G)+1.*

*Proof:* Notice that the vertex *x* does not identify any pair of vertices of *G* unless such pair contains the vertex *x* itself. In this sense, if *x* is not a twin, then *x* does not belong to any metric basis of *G*, and so, it is a void vertex. Thus, from Remark 2.3, we have that mmd(G)≥dim(G)+1. In addition, since *x* is universal, for any metric basis *S* of *G*, its complement is connected. Therefore, from Remark 2.4 we obtain the desired equality. ◻

The remark above has some consequences for some graph classes.

**Corollary 2.7.** *For any graph G of maximum degree at most* |V(G)|−2, $\text{mmd}(G+K_{1})=\dim(G+K_{1})+1$. In particular, this follows for wheels *W*_*n*_ *and fans F*_*n*_.

From Remarks 2.4 and 2.6, it can be noted that the difference between the metric dimension and the mobile metric dimension of graphs is one. We next show that this difference can be also arbitrarily large. To this end, we consider some necessary constructions. We first introduce the following family ℱ of graphs *G*_*k*_ for k≥3.

For each integer k≥3, *G*_*k*_ is a graph of order *k* + 2^*k*^ such that $V(G_{k})=A\cup B$, where A={1,…,k} and *B* is formed by all the possible subsets of the set {1,…,k} (clearly |*B*| = 2^*k*^), that is $B=\{A_{i}\;:\;A_{i}\subseteq A\}$. The edge set of *G*_*k*_ is specified as follows:

- each of the sets *A* and *B* induces a clique in *G*_*k*_;
- a vertex i∈A is adjacent to every vertex $A_{j}\in B$ such that $i\in A_{j}$.

**Fig 2:**
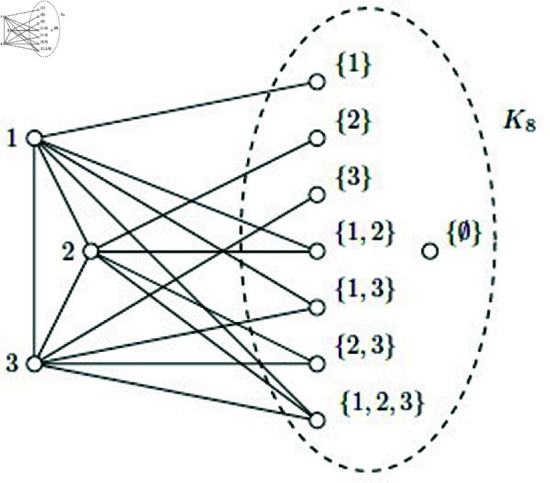
The graph ${\text{G}}_{3}$.

See Fig 2 for the graph *G*_3_ and the labeling of its vertices.

The next known result is necessary for our purposes.

**Theorem 2.8.** [4] *For any connected graph G of order n and diameter d, dim(G)≥f(n,d), where f(n,d) represents the least positive integer k such that $k+d^{k}\geq n$.*

**Lemma 2.9.** *For each k≥3, let $G_{k}\in ℱ$. Then,*

(i) $\dim(G_{k})=k$;
(ii) *if S is a resolving set of G*_*k*_ *such that* |S∩A|<k, *then* $|S|\geq 2^{k-1}+1$; *and*
(iii) *the set A is the unique metric basis of G*_*k*_.

*Proof:* We shall use the notation of the set of vertices of *G*_*k*_ used in its definition.

(i) Since for any two vertices $A_{i},A_{j}\in B$ it follows $N_{G_{k}}(A_{i})\neq N_{G_{k}}(A_{j})$, we deduce that $A_{i},A_{j}$ are identified by a vertex of *A*. Thus, *A* is a resolving set for *G*_*k*_, and so, $\dim(G_{k})\leq |A|=k$. Since *G* has diameter 2 and order 2^*k*^ + *k*, from Theorem 2.8, we deduce the equality.

(ii) Assume |S∩A|<k and let $S^{\prime }=A\cap S$. If $S^{\prime }=\emptyset$, then we deduce that $|S|=2^{k}-1\geq 2^{k-1}+1$ because *B* induces a clique, and any two vertices of B⧵S are not identified by vertices in *B*. Thus, we may assume that $S^{\prime }\neq \emptyset$. We now notice that a vertex ℓ∈A is the only vertex that identifies all the pair of vertices $A_{i},A_{j}\in B$ such that (w.l.g.) $A_{i}=A_{j}\cup \{\ell \}$. This means that for every vertex ℓ∈A such that ℓ∉S, there are $2^{\text{k}-1}$ distinct pairs of vertices in *B* which are not identified by the vertices of $S^{\prime }$. Since any pair of vertices $A_{i},A_{j}$ of *B* are also not identified by any vertex of *B* except themselves, we deduce that at least one vertex from each of these $2^{\text{k}-1}$ distinct pairs must be in $S^{\prime }$, that is $|S\cap B|\geq 2^{k-1}$. Consequently, $|S|\geq |S^{\prime }|+|S\cap B|\geq 2^{k-1}+1$.

(iii) By (i), if *S* is a metric basis of *G*_*k*_, then |S|=k. By (ii), if *S* is a resolving set such that S≠A, then $|S|\geq 2^{k-1}\;+\;1$, and so, *S* is not a metric basis. Thus, it must happen *S* = *A*, and so there is a unique metric basis in *G*_*k*_, which is *A*. ◻

Now, in order to prove that the difference between the metric dimension and the mobile metric dimension of graphs can be also arbitrarily large, we need the following construction. For an integer q≥2, we consider *q* copies of a graph $G_{k}\in ℱ$ for some k≥3. We next add all the possible edges between vertices belonging to the set *A* in each of the *q* copies of the graph *G*_*k*_. We denote the constructed graph as *G*_*q*,*k*_. Note that the union of the copies of the sets *A* induces a clique in *G*_*q*,*k*_. The next properties of such graphs can be readily observed, based on the fact that pairs of vertices of *G*_*q*,*k*_ belonging to a same copy of *A*, or of *B*, in one copy of *G*_*k*_ are only identified by vertices inside of such copy of *G*_*k*_ used to construct *G*_*q*,*k*_.

**Lemma 2.10.** *For any k≥3 and q≥2,*

(i) $\dim(G_{q,k})=k\cdot q$;
(ii) *if S is a resolving set of G*_*q*,*k*_ *such that* |S∩A|<k for some set A of some copy of *G*_*k*_ *in G*_*q*,*k*_, *then* $\left|S|\geq q(2^{k-1}+1\right)$; *and*
(iii) *the union of the sets A of each copy of G*_*k*_ *in G*_*q*,*k*_ *is the unique metric basis of G*_*q*,*k*_.

We might recall that the unique metric basis of *G*_*q*,*k*_ has the property that its complement does not induce a connected graph. This fact is a key point in our next result.

**Theorem 2.11.** *For any integer q≥2, there exist a graph G such that mmd(G)−dim(G)≥q.*

*Proof:* To prove our claim, we consider a graph *G*_*q*,*k*_. From Lemma 2.10 (i) and (iii), we know that $\dim(G_{q,k})=k\;\cdot \;q$ and that the union of the sets *A* of each copy of *G*_*k*_ in *G*_*q*,*k*_ is the unique metric basis of *G*_*q*,*k*_. Let $S^{\prime }$ be a set of vertices of *G*_*q*,*k*_ containing its unique metric basis together with exactly one vertex, say *A*_*j*_, from each set *B* of every copy of *G*_*k*_ in *G*_*q*,*k*_. Clearly, $|S^{\prime }|=k\cdot q+q$. Since the vertex *A*_*j*_ from each copy of *G*_*k*_ in *G*_*q*,*k*_ can visit all the vertices of *B* in its corresponding copy through a sequence of valid resolving movements, we obtain that $S^{\prime }$ is a mobile resolving set, and so, $\text{mmd}(G_{q,k})\leq |S^{\prime }|=k\cdot q+q$.

On the other hand, let *S* be a mobile metric basis of *G*_*q*,*k*_, and suppose that |S|<k·q+q=q(k+1). Since *G*_*q*,*k*_ has *q* copies of *G*_*k*_, by the pigeon hole principle, there must be a copy of *G*_*k*_, say $G_{k}^{<}$, in *G*_*q*,*k*_ such that it contains less than k+1 elements of *S*, that is at most *k* vertices of *S*. Indeed, $G_{k}^{<}$ cannot have less than *k* vertices of *S*, because then we would find a pair of vertices which is not identified by *S*, which is not possible. Thus, there must be exactly *k* vertices of *S* in $G_{k}^{<}$. Let ${\text{S}}^{*}$ be the set of vertices of *S* in $G_{k}^{<}$. We moreover notice that ${\text{S}}^{*}$ must be equal to the set *A* of $G_{k}^{<}$. Now, in order to visit a vertex *A*_*j*_ of *B* in $G_{k}^{<}$, we cannot use other vertices of *S* from other copies of *G*_*k*_, because every path between *A*_*j*_ and other vertices of *S* not in $G_{k}^{<}$ contains a vertex of ${\text{S}}^{*}$ in the way. Hence, vertices of *B* in $G_{k}^{<}$ need to be visited by one vertex of the set ${\text{S}}^{*}$. However, in such setting, no vertex of ${\text{S}}^{*}$ can make a valid resolving movement because if we move one vertex of ${\text{S}}^{*}$ to a neighbor in *B*, then there are at least $2^{k-1}\geq 2$ distinct pairs which are not identified by *S*, and this is not possible. Consequently, vertices of *B* in the copy $G_{k}^{<}$ cannot be visited by any vertex of *S*, which is a contradiction. Therefore, mmd(G)=|S|≥k·q+q, which gives the equality mmd(G)=k·q+q. Finally, since $\dim(G_{q,k})=k\cdot q$, our desired claim follows. ◻

Based on all the results from this section, one could think that the opposite of Remark 2.3 might be satisfied. Namely, if mmd(G)≥dim(G)+1 for a graph *G*, then *G* has a void vertex. We next show this is not the case, if we for instance, consider the case of the prism of an odd cycle. It is known from [3] that for any integers r≥2 and s≥3,


$$\dim(P_{r}□C_{n})=\{\begin{array}{ll} 2; & \text{if }s\;\text{is odd},\\ 3; & \text{if }s\;\text{is even}. \end{array}$$

Notice that the prism graph of a cycle *C*_*n*_ is precisely the Cartesian product $P_{2}\;◻\;C_{n}$. Moreover, it is also possible to check that $P_{2}\;◻\;C_{n}$ has no void vertex since it is a vertex transitive graph. As we next show, if *n* is odd, then $\text{mmd}(P_{2}\;◻\;C_{n})=3>2+1=\dim(P_{2}\;◻\;C_{n})+1$.

**Proposition 2.12.** *If n≥3 is an odd integer, then $\text{mmd}(P_{2}\;◻\;C_{n})=3$.*

*Proof:* From [3] it is known that $\dim(P_{2}\;◻\;C_{n})=2$. Let $S=\{(u,v),(u^{\prime },v^{\prime })\}$ be a metric basis of $P_{2}\;◻\;C_{n}$. Suppose $u\neq u^{\prime }$. Namely the two vertices $\left(u,v),(u^{\prime },v^{\prime }\right)$ belong to different copies of the cycle *C*_*n*_ in $P_{2}\;◻\;C_{n}$. If $\left(u,v),(u^{\prime },v^{\prime }\right)$ are adjacent, then $v=v^{\prime }$ and for the two neighbors of *v* in *C*_*n*_, say $v_{1},v_{2}$, it is satisfied that $d_{P_{2}\;◻\;C_{n}}((u,v_{1}),(u,v))=1=d_{P_{2}\;◻\;C_{n}}((u,v_{2}),(u,v))$ and $d_{P_{2}\;◻\;C_{n}}((u,v_{1}),(u^{\prime },v))=2=d_{P_{2}\;◻\;C_{n}}((u,v_{2}),(u^{\prime },v))$. Thus, $\left(u,v_{1}\right)$ and $\left(u,v_{2}\right)$ are not identified by *S*, which is a contradiction.

Assume now $\left(u,v),(u^{\prime },v^{\prime }\right)$ are not adjacent. Hence, by the structure of the graph $P_{2}\;◻\;C_{n}$, there are (at least) two completely disjoint shortest $\left(u,v),(u^{\prime },v^{\prime }\right)$-paths in $P_{2}\;◻\;C_{n}$ of length at least two. Thus, the two neighbors of (*u*,*v*) from two of such disjoint paths are neither by identified by (*u*,*v*) nor by $\left(u^{\prime },v^{\prime }\right)$, which is not possible. Therefore, if $S=\{(u,v),(u^{\prime },v^{\prime })\}$ is a metric basis of $P_{2}\;◻\;yC_{n}$, then it must happen $u=u^{\prime }$, or equivalently, the two vertices of *S* must belong to a same copy of a cycle in $P_{2}\;◻\;C_{n}$.

In consequence, if there is a set of robots placed at a resolving set of $P_{2}\;◻\;C_{n}$, in order to visit all the vertices of $P_{2}\;◻\;C_{n}$ by a sequence of valid resolving movements, then such resolving set cannot be a metric basis, due to the required structure of any metric basis of $P_{2}\;◻\;C_{n}$. Thus, $\text{mmd}(P_{2}\;◻\;C_{n})\geq 3$.

On the other hand, it can be readily seen that any metric basis of $P_{2}\;◻\;C_{n}$ together with any other vertex not in such metric basis is a mobile resolving set of $P_{2}\;◻\;C_{n}$. Therefore, $\text{mmd}(P_{2}\;◻\;C_{n})\leq 3$ and the equality follows. ◻

By using similar techniques as in the proof above, we can compute the value of $\text{mmd}(P_{r}\;◻\;C_{n})$ for any r≥3 and *n* odd. However, for this case (r≥3), the result follows directly from Remark 2.3, since in such situation, $P_{r}\;◻\;C_{n}$ has void vertices. Anyway, we next state the result without proof.

**Proposition 2.13.** *If r≥3 and n≥3 is an odd integer, then $\text{mmd}(P_{r}\;◻\;C_{n})=3$.*

## 3 Bounds and extremal cases

We continue our exposition with the following bounds. We recall that the *diameter* of a graph is the largest possible distance between any two vertices of the graph.

**Remark 3.1.** *For any graph G of order n≥3 and diameter d≥2, mmd(G)≤n−d+1.*

*Proof:* Let $v_{1}v_{2}\cdots v_{d+1}$ be a diametral path in *G*. We claim that the set $S=V(G)⧵\{v_{2},\ldots ,v_{d}\}$ forms a mobile resolving set for *G*. To see this, we need to notice that the vertex $v_{1}$ identifies all the vertices $v_{2},\ldots ,v_{d+1}$. Thus, the robot located at the vertex $v_{d+1}$ can visit all the vertices of the set $\left\{v_{2},\ldots ,v_{d}\right\}$ by the sequence of resolving movements $v_{d+1}⇝v_{d}⇝\cdots ⇝v_{2}$. Therefore, mmd(G)≤|S|=n−d+1 as desired. ◻

It is now a kind of folklore result that a graph *G* has metric dimension one if and only if the graph is a path. In this sense, due to (1), it is clear that if *G* satisfies that mmd(G)=1, then *G* must be a path. However, only a path on two vertices holds such fact. This gives sense to the lower bound of our next basic result.

**Remark 3.2.** *For any graph G of order n≥3, 2≤mmd(G)≤n−1.*

*Proof:* The upper bound trivially follows by the fact any set of vertices of *G* with cardinality *n*–1 is a resolving set of *G*. On the other hand, since any mobile resolving set is also a resolving set, we have that mmd(G)≥dim(G)≥1. Now, if mmd(G)=1, then dim(G)=1 and *G* must be a path. If *G* is a path, then any metric basis of *G* is formed by exactly one of its leaves. But then, no robot placed at a leaf of a path of order at least 3 can make a valid resolving movement. This means that, in order to have a mobile resolving set in such a path, we need at least two vertices. Therefore, mmd(G)≥2, which gives the lower bound. ◻

We next center our attention on the graphs attaining the limit values in the trivial bounds above. To this end, we say that ℋ is the family of graphs *H*_*s*,*t*_ that can be obtained from the join of two graphs *H* and $H^{\prime }$ of orders *s* and *t* (*s* or *t* can be zero), respectively, such that $H,H^{\prime }$ are cliques or empty graphs. Notice that for instance complete graphs *K*_*n*_ (*s* = *n* and *t* = 0) and complete bipartite graphs *K*_*s*,*t*_ are graphs of the family ℋ.

**Theorem 3.3.** *Let G be a graph of order n. Then mmd(G)=n−1 if and only if G∈ℋ.*

*Proof:* First, it can be easily observed that if G∈ℋ, then mmd(G)=n−1. On the other hand, assume mmd(G)=n−1. From Remark 3.1, we deduce that *G* has diameter at most 2. If *G* has diameter 1, then *G* is a complete graph, and so G∈ℋ. Hence, we consider *G* has diameter 2. Let *A* be an independent set of largest cardinality in *G*. Notice that |A|≥2, for otherwise *G* is complete. Also, let B=V(G)⧵A. If |B|=1, then *G* is a star, namely, a complete bipartite graph *K*_1,*n*−1_, and so, G∈ℋ. In consequence, we may assume |A|≥2 and |B|≥2.

Now, suppose *B* does not induce neither a clique nor an empty graph. Let u,v∈B such that uv∉E(G) and let $u^{\prime },v^{\prime }\in B$ such that $u^{\prime }v^{\prime }\in E(G)$. Hence, we must have |B|≥3. Consider now the subgraph $G^{\prime }$ induced by the vertices $u,v,u^{\prime },v^{\prime }$. Note that $G^{\prime }$ has at least three vertices. Moreover, there must be two vertices $x,y\in V(G^{\prime })$ (which might be adjacent or not), such that one of them, say *x*, has a neighbor, say $z\in V(G^{\prime })$, which is not a neighbor of *y*. We have two situations.

Case 1: *x*,*y* are not adjacent. Let S=V(G)⧵{x,y}. Observe that z∈S and that *z* identifies the pair *x*,*y*. Thus, *S* is a resolving set of *G*. Now, let $x^{\prime }$ be a neighbor of *x* in *A*, which exists because *A* is an independent set of largest cardinality in *G*. Then, the robot placed in $x^{\prime }$ moves to *x*, and let $S^{\prime }=S⧵\{x^{\prime }\}\cup \{x\}$. Since *x*,*y* are not adjacent, we have that $x\in S^{\prime }$ identifies the pair $x^{\prime },y$ and so, $S^{\prime }$ is also a resolving set. Now, we turn back this previous movement, that is, consider $S^{\prime \prime }=S^{\prime }⧵\{x\}\cup \{x^{\prime }\}$, which is indeed the same as *S*. Next we perform a similar movement but using *y* and a neighbor $y^{\prime }\in A$ instead of *x* and $x^{\prime }$ and consider the set $x^{\prime }$. By similar arguments, $x^{\prime }$ identifies the pair $x,y^{\prime }$ and so, $x,y^{\prime }$ is also a resolving set. Thus, we have developed a sequence of valid resolving movements to visit all the vertices of *G*. Therefore, *S* is a mobile resolving set of cardinality *n*–2, which is not possible.

Case 2: *x*,*y* are adjacent. Let again $x^{\prime },y^{\prime }\in A$ be neighbors of *x*,*y*, respectively. If $x^{\prime }\neq y^{\prime }$, then the set S=V(G)⧵{x,y} taken as before is a mobile resolving set, by using the following two sequences of movements $x^{\prime }⇝x⇝x^{\prime }$ and also $y^{\prime }⇝y$. This is a contradiction since *S* has cardinality *n*–2. Hence, we may assume that *x*,*y* have exactly one neighbor in *A*, and that such neighbors coincide, say *w* is such neighbor. Since |A|≥2, let $w^{\prime }\in A⧵\{w\}$. Clearly, $w^{\prime }$ is not adjacent to *x* nor to *y*. Since *G* is connected and *A* is independent, $w^{\prime }$ must have a neighbor $z^{\prime }\in B$. Hence, the vertices $w,w^{\prime }$ are identified by any of $x,y,z^{\prime }$ and so, $S=V(G)⧵\{w,w^{\prime }\}$ is a resolving set. Now, we make the following sequence of movements:

- x⇝w, and the set $S^{\prime }=S⧵\{x\}\cup \{w\}$ is a resolving set since *w* identifies the pair $x,w^{\prime }$.
- w⇝x and the set $S^{\prime \prime }=S^{\prime }⧵\{w\}\cup \{w\}=S$ is a resolving set.
- $z^{\prime }⇝w^{\prime }$, and the set $S^{\prime \prime }=S^{\prime }⧵\{z\}\cup \{w\}=S$ is a resolving set since $w^{\prime }$ identifies the pair $z^{\prime },w$.

Thus, we deduce that *S* is a mobile resolving set of cardinality *n*–2, which is again not possible.

Therefore, the contradictions from the two cases above allow to deduce that *B* induces either a clique or an empty graph. In this sense, we next consider these two possible situations.

Case 3: *B* induces a clique. If every vertex of *A* is adjacent to every vertex of *B*, then G∈ℋ. In this sense, suppose there is a∈A and b∈B such that ab∉E(G). Let $b^{\prime }\in B$ such that $ab^{\prime }\in E(G)$ (note that such $b^{\prime }$ exists because *A* is independent and *G* is connected). Also notice that $bb^{\prime }\in E(G)$ because *B* induces a clique. Let $S=V(G)⧵\{a,b^{\prime }\}$. It is clear that such *S* is a resolving set, since $a,b^{\prime }$ are identified at least by b∈S. Now, consider the robot placed in *b* moves to $b^{\prime }$, and let $S^{\prime }=S⧵\{b\}\cup \{b^{\prime }\}$. Now, *a*,*b* are not identified by $b^{\prime }$, but they are identified by some neighbor, say $a^{\prime }\in A$, of *b* in $S^{\prime }\cap A$. Such $a^{\prime }$ exists because *b* needs to have at least one neighbor in *A* (and such neighbor is not *a*), since *A* is a largest independent set. Thus, $S^{\prime }$ is also a resolving set. Next, the robot placed in $b^{\prime }$ moves to *a*, and let $S^{\prime \prime }=S^{\prime }⧵\{b^{\prime }\}\cup \{a\}$. Clearly, now $S^{\prime \prime }$ is also a resolving set since *a* identifies $b,b^{\prime }$. As a consequence, we obtain that *S* is a mobile resolving set of cardinality *n*–2, which is a contradiction. Thus, every vertex of *A* is adjacent to every vertex of *B*, which means G∈ℋ.

Case 4: *B* induces an empty graph. If every vertex of *A* is adjacent to every vertex of *B*, then *G* is a complete bipartite graphs, and so G∈ℋ. Hence, as in the previous case, suppose there is a∈A and b∈B such that ab∉E(G), and also similarly, let $a^{\prime }\in A$ and $b^{\prime }\in B$ such that $ab^{\prime },a^{\prime }b\in E(G)$. Since $a,a^{\prime }$ are identified by *b*, we have that $S=V(G)⧵\{a,a^{\prime }\}$ is a resolving set for *G*. Now, it can be readily find two sequences of valid resolving movements that allows to claim *S* is a mobile resolving set. The sequences are $b⇝a^{\prime }⇝b$ and $b^{\prime }⇝a$. This is a contradiction since *S* has cardinality *n*–2.

This final contradiction completes the proof of the theorem. ◻

Now, in order to consider some partial results concerning a possible characterization of the graphs achieving the equality in the lower bound of Remark 3.2, we need the following construction. Let 𝒥 be the family of graphs *G*_*r*,*t*_, with r≥3 and t≥2, obtained from a cycle *C*_*r*_ and a path *P*_*t*_ by identifying one vertex of *C*_*r*_ with a leaf of *P*_*t*_.

**Proposition 3.4.** *Let $G_{r,t}\in 𝒥$. Then $\text{mmd}(G_{r,t})=2$ if and only if r is odd.*

*Proof:* Let $C_{r}=v_{0}v_{1}\cdots v_{r-1}v_{0}$, $P_{t}=u_{1}u_{2}\cdots u_{t}$ and assume $v_{0}$ is identified with *u*_1_ in *G*_*r*,*t*_, hence to simplify the notation let *w* be the vertex obtained by the identification of $v_{0}$ and *u*_1_.

(⇐) Let *r* be an odd integer. We claim that the set $S=\{v_{r-1},v_{1}\}$ is a mobile resolving set of *G*_*r*,*t*_. We first observe that *S* is a resolving set. Let $x,y\in V(G_{r,t})$ and consider some situations.

- If $x,y\in V(P_{t})$, then they are clearly identified by $v_{r-1}$ and by $v_{1}$.
- If $x,y\in V(C_{r})$, then they are clearly identified by $v_{r-1}$ and by $v_{1}$, since r≠4.
- If $x\in V(P_{t})$, $y\in V(C_{r})$ and $d_{G_{r,t}}(x,v_{r-1})=d_{G_{r,t}}(y,v_{r-1})$, then clearly $y\neq w=v_{0}$. Moreover, since $d_{G_{r,t}}(x,v_{r-1})=d_{G_{r,t}}(x,w)\;+\;1=d_{G_{r,t}}(x,v_{1})$ and $d_{G_{r,t}}(y,v_{r-1})\neq d_{G_{r,t}}(y,v_{1})$ (because r≠4 and $y\neq v_{0}$) we deduce that *x*,*y* are identified by $v_{1}$.

As a consequence of the items above, we conclude that *S* is a resolving set. Let us now describe a sequence of valid resolving movements in order to visit all the vertices of *G*_*r*,*t*_ beginning with vertices of the set *S*. To this end, we use the following fact that can be readily checked.

**Claim A:** Any two vertices of an odd cycle form a resolving set of such cycle.

Now, let us consider the following sequence of movements of the robots in *S*: $v_{1}⇝v_{2}⇝\cdots ⇝v_{\lfloor r/2\rfloor }$. Notice that if *r* = 3, then no movement is made and we are done. Hence, we may assume r≥5. At each step notice that the set of robots is given by $S_{j}=\{v_{r-1},v_{j}\}$ with j∈{1,…,⌊r/2⌋}. We shall prove that every *S*_*j*_ is a resolving set for j≥2 since *j* = 1 has already been considered. By **Claim A**, any two vertices of the cycle are identified by at least one of the vertices from each *S*_*j*_. Also, any two vertices of the path *P*_*t*_ are identified by every vertex of these sets. It remains to consider a vertex *x* from *P*_*t*_ different from $u_{1}=w=v_{0}$ and a vertex $y=v_{i}$ (for some i≠0) from the cycle. Suppose that

$$d_{G_{r,t}}(x,v_{r-1})=d_{G_{r,t}}(x,w)+1=d_{G_{r,t}}(y,v_{r-1})$$ (4)

and

$$d_{G_{r,t}}(x,v_{j})=d_{G_{r,t}}(x,w)+d_{G_{r,t}}(w,v_{j})=d_{G_{r,t}}(y,v_{j})$$ (5)

where $v_{j}\in S_{j}$. Thus, in consequence with this, it must happen that *y* is a vertex of the path $P^{\prime }=v_{j}v_{j+1}\cdots v_{r-1}$. Moreover, the shortest $y,v_{j}$-path does not intersect with the shortest $y,v_{r-1}$-path (except in *y*); nor with the $w,v_{j}$-path; nor with the edge $wv_{r-1}$. According to these comments, it must occur that

$$d_{G_{r,t}}(w,v_{j})+d_{G_{r,t}}(y,v_{j})+d_{G_{r,t}}(y,v_{r-1})+1=r.$$ (6)

On the other hand, from (4) and (5) we deduce that


$$d_{G_{r,t}}(y,v_{r-1})-1=d_{G_{r,t}}(y,v_{j})-d_{G_{r,t}}(w,v_{j}).$$

Therefore, by summing up the last equality with (6), we obtain that $2d_{G_{r,t}}(y,v_{r-1})\;+\;2d_{G_{r,t}}(w,v_{j})=r$. Hence, *r* is an even integer, which is a contradiction with our assumption. Thus, it holds that *x*,*y* are identified by $v_{r-1}$ or by $v_{j}$, and so, each *S*_*j*_ is a resolving set. Consequently, $v_{1}⇝v_{2}⇝\cdots ⇝v_{\lfloor r/2\rfloor }$ is a sequence of valid resolving movements.

By the same arguments as above, also the movements $v_{\lfloor r/2\rfloor }⇝v_{\lfloor r/2\rfloor -1}⇝\cdots ⇝v_{1}$ are valid resolving movements as well, and by symmetry also the movements $v_{r-1}⇝v_{r-2}⇝\cdots ⇝v_{\lceil r/2\rceil }$ are such.

Consequently, we have now visited all the vertices of the cycle but *w*. At this point notice that the robots are placed at the resolving set $S^{\prime }=\{v_{1},v_{\lceil r/2\rceil }\}$. We now make the movement $v_{1}⇝v_{0}=w=u_{1}$, and shall prove that the set $S^{\prime \prime }=\{w,v_{\lceil r/2\rceil }\}$ is a resolving set.

By **Claim A**, any two vertices of the cycle are identified by at least one of the vertices from $S^{\prime \prime }$. Also, any two vertices of the path *P*_*t*_ are identified by the two vertices of $S^{\prime \prime }$. We need to consider a vertex *x* from *P*_*t*_ and a vertex *y* from the cycle. Since *w* and $v_{\lceil r/2\rceil }$ are diametral vertices and *r* is odd, it can be readily observed that if $d_{G_{r,t}}(x,w)=d_{G_{r,t}}(y,w)$, then $d_{G_{r,t}}(x,v_{\lceil r/2\rceil })\neq d_{G_{r,t}}(y,v_{\lceil r/2\rceil })$. Thus, $S^{\prime \prime }$ is a resolving set, and so $v_{1}⇝w$ is a valid resolving movement.

Now, in order to complete the proof of our initial claim (that $S=\{v_{r-1},v_{1}\}$ is a mobile resolving set of *G*_*r*,*t*_), we make the following movements $w=u_{1}⇝u_{2}⇝u_{t}$ (notice that we are using the set $S^{\prime \prime }=\{w,v_{\lceil r/2\rceil }\}$). It can be also easily verified that at each step the set $S_{j}^{\prime \prime }=\{u_{j},v_{\lceil r/2\rceil }\}$, with j∈{2,…,t}, is a resolving set. Thus, $w=u_{1}⇝u_{2}⇝u_{t}$ are valid resolving movements, which allows to finally conclude that $S=\{v_{r-1},v_{1}\}$ is a mobile resolving set of *G*_*r*,*t*_, and the proof of this implication is completed.

(⇒) Assume now $\text{mmd}(G_{r,t})=2$. Suppose that *r* is even. We shall prove that the vertex $v_{0}=u_{1}=w$ is a void vertex of *G*_*r*,*t*_. For the contrary, suppose there is a metric basis *R* such that w∈R and let z∈R⧵{w}. Clearly, *z* cannot belong to the path *P*_*t*_ since then *R* is not a resolving set. Thus, *z* must be in the cycle *C*_*r*_. Hence, at least one of the vertices $v_{1}$ or $v_{r-1}$, say $v_{1}$, is not in *R*. Consider the pair of vertices $u_{2},v_{1}$. In order to identify such pair (since they have the same distance to w∈R), it must happen that $z\notin \{v_{r-1},v_{r-2},\ldots ,v_{r/2+1}\}$. This means that also $v_{r-1}\notin R$. Thus, to identify the pair $u_{2},v_{r-1}$ it must similarly happen that $z\notin \{v_{1},v_{2},\ldots ,v_{r/2-1}\}$. Consequently, *z* must be equal to $v_{r/2}$, but then there are several pairs of vertices (for instance the two neighbors of $w=v_{0}$ in the cycle) that are not identified by *R*, which is a contradiction. Thus, *w* cannot belong to any metric basis, and so it is a void vertex of *G*_*r*,*t*_. Since *G*_*r*,*t*_ is not a path, it is satisfied that $\dim(G_{r,t})\geq 2$. Therefore, by using Remark 2.3 we deduce that $\text{mmd}(G_{r,t})\geq 3$, a contradiction with the statement. This allows to conclude that *r* needs to be odd and the proof is completed. ◻

The result above, Corollary 2.5 and Proposition 2.1 allow to observe that there are several different classes of graphs *G* for which $\text{mmd}(G_{r,t})=2$. Moreover, there are also some other distinct graphs satisfying this property. To see this, consider for instance a cycle of order three with a pendant vertex added to each vertex (this graph is indeed the corona of *C*_3_). It can be readily observed (using the symmetry of such graph) that any set of two vertices of the cycle *C*_3_ is a mobile resolving set. In this sense, it is maybe worthy of considering characterizing all the graphs *G* with $\text{mmd}(G_{r,t})=2$.

### 3.1 The mobile metric dimension versus the resolving number

The *resolving number* res(G) of a graph *G* was first defined in [5] as the smallest integer *k* such that every set of vertices of *G* of cardinality at least *k* is a resolving set for *G*. The parameter has been studied in a few other articles, and among them, we remark [11], where the authors proved that finding the resolving number of graphs can be done in polynomial time. To this end, given two vertices x,y∈V(G), let


$$R_{G}(x,y)=\{v\in V(G)\;:\;d_{G}(x,v)\neq d_{G}(y,v)\}\;\text{ and }\;{\overset{―}{R}}_{G}(x,y)=V(G)⧵R_{G}(x,y).$$

It was shown in [11] that for any graph *G* of order *n*,


$$\text{res}(G)=\max_{x,y\in V(G)}|{\overset{―}{R}}_{G}(x,y)|+1,$$

and that such quantity can be computer in $𝒪(n^{3})$ time. This result turns to be very useful for our purposes of bounding the mobile metric dimension of graphs. That is next stated.

**Proposition 3.5.** *For any graph G, mmd(G)≤res(G).*

*Proof:* If S⊂V(G) is a set of cardinality res(G), then it is a resolving set. Thus, a movement from a vertex of *S* to any of its neighbors not in *S* (if they exist) is a valid resolving movement, because any set of cardinality res(G) is a resolving set of *G*. Thus, *S* is a mobile resolving set and the bound follows ◻

The bound above is tight for several graphs classes. For instance, if *G* is a path of order at least three or a cycle of odd order, then it is satisfied that mmd(G)=res(G)=2. Complete bipartite graphs *K*_*r*,*s*_ are other graphs achieving the previous equality since $\text{mmd}(K_{r,s})=\text{res}(K_{r,s})=r+s-1$. On the other hand, since dim(G)≤mmd(G) for any graph *G*, by using Proposition 3.5 we deduce the following result.

**Remark 3.6.** *If G satisfies that dim(G)=res(G), then dim(G)=mmd(G)=res(G).*

Although there are several graphs *G* for which the equality mmd(G)=res(G) holds, we observe that the difference between such parameters can be arbitrarily large. For instance, consider the grid graph $P_{r}\;◻\;P_{s}$. From Corollary 2.5, $\text{mmd}(P_{r}\;◻\;P_{s})=3$. However, $\text{res}(P_{r}\;◻\;P_{s})\geq (r-1)(s-1)+1$. To see this, notice that if $P_{r}=u_{1}u_{2}\cdots u_{r}$ and $P_{s}=v_{1}v_{2}\cdots v_{s}$, then the set of vertices $S=\{u_{1},\ldots ,u_{r-1}\}\times \{v_{1},\ldots ,v_{s-1}\}$ is not a resolving set of $P_{r}\;◻\;P_{s}$, since the two vertices $\left(u_{r},v_{s-1}),(u_{r-1},v_{s}\right)$ are not identified by *S*.

## 4 Concluding remarks

We have considered in this work a model for mobile robots in a network keeping the property of uniquely identifying all the vertices of the network while the robots move from one point to another, and with the requirement of visiting all the nodes of the networks at least once. We have given several combinatorial properties of smallest sets of nodes achieving this properties. As usual, there are several open questions that might deserve attention to be widely explored. We next remark a few of them which are of interest from our point of view.

- The problem of finding a mobile resolving set depends on finding the best possible way for moving the robots from the mobile resolving set. In this sense, it is of interest to develop efficient strategies for such movements of the robots.
- Which is the complexity of finding the mobile metric dimension of graphs? Is it polynomial to check on whether a given set of vertices is indeed a mobile resolving set?
- Is there any heuristic that can be developed to find or approximate the value of the mobile metric dimension of graphs?
- From Remark 2.3 we have that if *G* has a void vertex, then mmd(G)≥dim(G)+1. From Proposition 2.12 there are also graphs without void vertices for which mmd(G)≥dim(G)+1. Can we characterize all the graphs *G* without void vertices and such that mmd(G)≥dim(G)+1?
- Based on Theorem 2.2, it seems of interest to complete the study of the mobile metric dimension of circulant graphs, or even more general, on vertex transitive graphs.
- In view of Proposition 3.5, it would be of interest characterizing the class of graphs *G* satisfying the equality mmd(G)=res(G).
- The mobile resolving sets of a graph frequently have the property that only one of its vertices is indeed “moving” through out all the vertices of the graph, while the remaining ones are those “keeping” the resolvability properties. This model could sometimes be not a realistic one. In this sense, as a future research line, it would be of interest to consider a model for mobile robots in networks (keeping some resolvability properties) in which all the robots visit all the vertices of the graph, by using the same rules as the mobile robots of this present investigation. On the other hand, one may also consider a variant of this problem in which not only one robot is moving at the same time.
- The edge metric dimension of graphs is a variant in which the edges of a graph are uniquely recognize by distances from a set of vertices of the graph (see [23]). It is also known that the metric and edge metric dimension of graphs are in general not comparable. In this sense, it might be of interest to consider a mobile version of the edge metric dimension, and among other contributions, check on whether the non-comparability properties remain between the mobile metric and edge metric dimensions of graphs.
